# Great prognosis of concurrent anti-GBM disease and IgA nephropathy in a young woman: A case report

**DOI:** 10.1097/MD.0000000000030686

**Published:** 2022-09-16

**Authors:** Fu Shaojie, Su Sensen, Huang Jingda, Wang Luyu, Zhang Fei, Yu Jinyu, Xu Zhonggao, Wu Hao

**Affiliations:** a Department of Nephrology, The First Hospital of Jilin University, Changchun, China.

**Keywords:** anti-glomerular basement membrane disease, corticosteroid, crescentic glomerulonephritis, immunoglobulin A nephropathy, plasmapheresis

## Abstract

The causal relationship between anti-glomerular basement membrane (anti-GBM) disease and immunoglobulin A (IgA) nephropathy is still unclear and cases of concurrent anti-GBM disease and IgA nephropathy are very rare, especially with a good prognosis and long-term follow-up. Here, we report a case of concurrent anti-GBM disease and IgA nephropathy. By using corticosteroids and cyclophosphamide in combination with plasmapheresis, the patient achieved a very good prognosis with complete normalization of renal function and complete disappearance of hematuria and proteinuria at the subsequent follow-up. To our knowledge, no previous case with such a long follow-up and such a good prognosis have been reported.

**Patient concerns:**

This case report describes a 26-year-old Chinese woman who presented with fever as the initial symptom, followed by dysmorphic hematuria, overt proteinuria and rapidly worsening renal function. Before admission, the patient received symptomatic supportive treatment such as intravenous albumin infusion, improvement of circulation, but the symptoms were not significantly improved.

**Diagnosis:**

Per the results of kidney biopsy, the patient was diagnosed with crescentic glomerulonephritis and anti-GBM disease with IgA nephropathy.

**Interventions:**

The key to obtain a good prognosis was the early application of corticosteroids and cyclophosphamide in combination with plasmapheresis to make the anti-GBM antibody turn negative quickly.

**Outcomes:**

After 2 weeks of therapy, the patients’ anti-GBM antibody turned negative and serum creatinine improved to a normal range. After 10 months, the patient’s proteinuria level reached complete remission. After 12 months, the patient’s hematuria had disappeared completely.

**Lessons:**

This case provides experience in the treatment of concurrent anti-GBM disease and IgA nephropathy and highlights the importance of early application of plasmapheresis and immunosuppressive therapy to obtain a good prognosis.

## 1. Introduction

Anti-glomerular basement membrane (anti-GBM) disease is a rare autoimmune condition characterized by linear deposition of immunoglobulin G along the GBM.^[1]^ Anti-GBM disease is mediated by circulating autoantibodies and its main autoantigen is the NC1 domain of the α3 chains of collagen IV on GBM.^[2]^ Clinically, it classically presents as rapidly progressive glomerulonephritis (RPGN), and histologically, it is closely associated with crescentic glomerulonephritis.^[3]^ The association between anti-GBM disease and anti-neutrophil cytoplasmic antibody (ANCA) is well known, and 21% to 47% of patients with anti-GBM disease are identified with coexisting ANCA-associated vasculitis.^[4]^ However, cases of concurrent anti-GBM disease and immunoglobulin A (IgA) nephropathy are very rare. Here, we report a new case of concurrent anti-GBM disease and IgA nephropathy admitted to our medical center and review all the cases published in PubMed to better understand the features of the disease.

## 2. Case presentation

A 26-year-old woman was admitted to the nephrology department of the first hospital of Jilin University with the presenting complaints of gross hematuria for 2 weeks, together with heavy proteinuria (10.2 g/24 h) and anti-basement membrane antibody positive. Two months before admission, the patient developed cough and expectoration, and the symptoms improved significantly after taking Chinese patent medicine (specific drugs are unknown). One month before admission, she developed fever, with the highest body temperature of 38°C. Ten days later foam urine was presented. And laboratory tests performed in a local hospital reported C-reactive protein 99.57 mg/L (the normal reference range: 0–10 mg/L), hematuria with a red blood cell (RBC) count of 1258 cells/μL (the normal reference range: 0–25 cells/μL), and 3 + protein for urinalysis. Routine blood test and procalcitonin were in the normal range and ultrasonogram showed normal-sized kidneys. She was given cefazolin and traditional Chinese medicine injection called Xiyanping Injection for fever and foam urine. Three days later, the condition worsened and gross hematuria was present. The patient was then transferred to another hospital, where he was tested for erythrocyte sedimentation rate 125 mm/h (the normal reference range: 0–20 mm/h), 24 h urinary protein 10.2 g/24 h (the normal reference range: 0–0.15 g/24 h), plasma albumin 23 g/L (the normal reference range: 35–55 g/L) and positive result of anti-GBM antibody. Due to the patient’s concern about side effects of immunosuppression, symptomatic supportive treatment such as intravenous albumin infusion, improvement of circulation, prevention and control of infection with cephalosporin antibiotics was given. After above treatment, the symptoms of fever, hematuria and proteinuria were not significantly improved. She had no history of drinking or smoking and no exposure to organic solvents and hydrocarbons. Her cardiac, pulmonary, and abdominal examinations were normal, with previously normal renal function and no significant past medical history.

As the symptoms remained unimproved, the patient was transferred to our hospital (2020.03.23). Upon admission, blood pressure was normal at 100 to 115/65 to 75 mm Hg, and no positive signs were found on physical examination, except for slight bilateral lower extremities edema. Blood tests showed a serum albumin level of 31 g/L, creatinine of 1.97 mg/dL (174.1 μmol/L), C-reactive protein of 93.4 mg/L and erythrocyte sedimentation rate of 95 mm/h. Urinalysis showed proteinuria of 3.4 g/d and hematuria of 1032 RBCs/high power field. The mycoplasma pneumoniae antibody was weakly positive and the influenza B virus antibody was positive. Serological tests for antinuclear antibodies, anti-double-strand DNA antibodies, anti-neutrophil cytoplasmic antibodies and anti-PLA2R antibodies were all negative. Elevated levels of IgA (4.61 g/L, the normal range is 0.71–3.85 g/L), complement C3 (1.53 g/L, the normal range is 0.8–1.2 g/L), complement C4 (0.41 g/L, the normal range is 0.1–0.4 g/L), and anti-GBM antibody (142 RU/mL) had also been found. In addition, serum immunofixation electrophoresis, blood glucose levels, tumor markers, and thyroid function were within normal ranges. There was no evidence of hepatitis virus, HIV, or syphilis infections. T-spot tuberculosis test result was also negative. Chest computed tomography and abdominal color doppler ultrasonography findings were normal.

**Figure 1.**
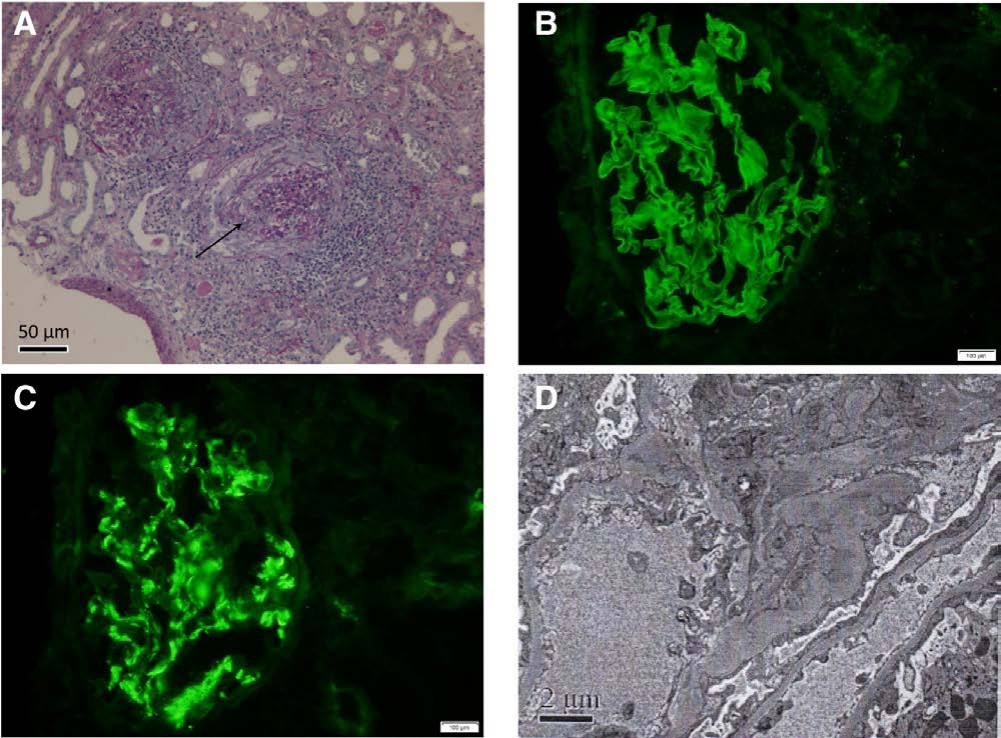
Pathological results of kidney biopsy. (A) Light microscopy (PASM, scale bars = 50 μm) of renal biopsy tissue revealed the formation of large crescents, the pathological manifestations were dominated by cellular crescents. (B) Representative photographs of immunofluorescence staining (Scale bars = 100 μm). Strong linear deposition for IgG along the glomerular capillary wall. (C) Representative photographs of immunofluorescence staining (Scale bars = 100 μm). IgA staining in clumps was found in mesangial areas. (D) Electron microscopic photograph of renal biopsy (Scale bars = 2 μm). Showing the electron-dense deposits in mesangial areas. IgA = immunoglobulin A, IgG = immunoglobulin G.

**Figure 2.**
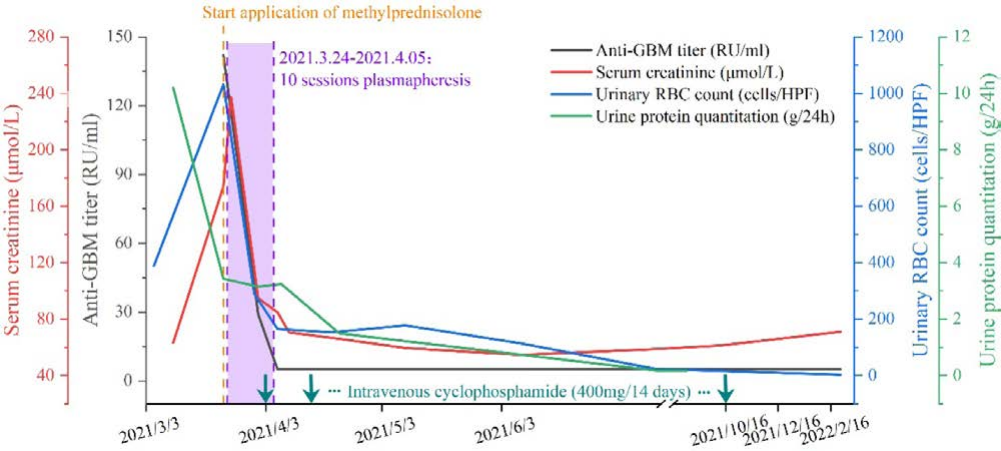
Changes in important test results during the course of the patient’s illness and follow-up.

Renal biopsy yielded a cortex containing 40 glomeruli with 1 global sclerosis, and mild proliferation of the mesangial areas was found. A total of 19 crescents were found, including 5 large-cellular that compressing glomerular capillary loop, with 7 Bowman’s capsules damaging to different degrees and fibroid necrosis of glomerular capillary walls. Tubular atrophy with luminal erythrocyte casts was observed (Fig. 1A). Immunofluorescence showed strong linear capillary loop staining for immunoglobulin G (Fig. 1B) and C3, together with mesangial staining for IgA (Fig. 1C). IgM, complementary C4 and Clq were not detected. Electron microscopy showed a mild increase in the mesangial matrix with lumpy electron-dense deposits in the mesangial space and extensive podocyte effacements were noted (Fig. 1D). Based on the above findings, a diagnosis of anti-GBM disease with IgA nephropathy was confirmed. The patient was treated with a pulse dose of intravenous methylprednisolone of 500 mg/day for 3 days followed by maintenance intravenous methylprednisolone at 40 mg/day. After 2 weeks, oral prednisone acetate (55 mg/day) was administered, tapered to 13.5 mg/day after 5 months and gradually discontinued after 12 months, according to the follow-up results. She underwent plasmapheresis for 10 sessions during hospitalization, until the anti-GBM antibody turned negative. Intravenous cyclophosphamide (400 mg/14 days) was also commenced, with a cumulative application of 6 g. In addition, she was treated with intravenous moxifloxacin (400 mg/day) and oral oseltamivir (75 mg/day) for 5 days because of mycoplasma and influenza B virus infection. As shown in Figure 2, after 2 weeks of therapy, laboratory examinations demonstrated the following results: urine test showed 165 RBCs/HPF, serum creatinine improved to a normal range, 24 h urine protein decreased to 3.24 g/24 h, anti-GBM antibody was negative, and both mycoplasma and influenza B virus antibodies were negative. After 10 months, the patient’s proteinuria level reached complete remission, urine tests showed 12 RBCs/HPF, serum creatinine remained within the normal range, and anti-GBM antibody was continually negative. After 12 months, the patient’s hematuria had disappeared completely.

## 3. Discussion

In this study, we report a rare case of anti-GBM disease accompanied with IgA nephropathy that presented with fever as the initial symptom, followed by dysmorphic hematuria, overt proteinuria and rapidly worsening renal function. Anti-GBM disease is a life-threatening, immune complex–mediated small vessel vasculitis.^[5]^ It is caused by antibodies reactive to intrinsic antigens in both the glomerular and alveolar basement membranes and commonly manifests as RPGN with or without diffuse alveolar hemorrhage.^[3]^ Anti-GBM disease is rare, with an estimated incidence of 1 to 2 cases per million population per annum.^[6,7]^ The correlation between anti-GBM disease and ANCA-associated vasculitis has been well documented in many studies, and 21% to 47% of patients with anti-GBM disease are identified with coexisting ANCA-associated vasculitis.^[4]^ However, concomitant anti-GBM disease with immune complex-mediated glomerulonephritis is rarely reported, among which membranous nephropathy is relatively common, while IgA nephropathy is even rarer.^[8]^ To better understand the clinical features of concomitant anti-GBM disease with IgA nephropathy, we reviewed and summarized all concurrent anti-GBM disease and IgA nephropathy cases published in PubMed (16 cases in total, including this case), as shown in Table 1.^[9–23]^

**Table 1:** Clinical and histological features of patients with concurrent anti-GBM disease and IgA nephropathy.

Case	Age (yr)/gender	Special medical history	Creatine before treatment (μmol/L)	Erythrocyturia	Proteinuria	Imageological examination		Renal biopsy		Anti-GBM antibody level	Treatment	Outcome
						Chest CT	Renal ultrasound	Crescent ratio	Immunofluorescence			
1^[9]^	31/F	No	287	4+	3.76 g/24 h	Pleural effusion and pachynsis pleurae bilaterally	Slight enlargement of both kidneys	82%	Linear capillary loop staining for IgG (3+) together with mesangial staining for IgA (3+), IgM (1+), and C3 (2+)	93.5 U/mL	Pulse dose of intravenous methylprednisolone/plasma exchange/sequential steroid combined with cyclophosphamide	Anti-GBM antibody turned negative after 29 d and serum creatinine was 375 μmol/L
2^[10]^	46/M	No	583.4	100/HPF	2.98 g/24 h	Normal	Normal sized kidneys	94%	Linear capillary loop staining for IgG together with mesangial staining for IgA and C3	214 U/mL	Pulse dose of intravenous methylprednisolone/sequential steroid monotherapy	Dialysis dependence
3^[11]^	24/M	HBV-infected	1387.9	30/HPF	7.04 g/24 h	Normal	Normal sized kidneys	58%	Strong linear capillary loop staining for IgG and C3 together with mesangial staining for IgA	Positive	Pulse dose of intravenous methylprednisolone and cyclophosphamide/plasma exchange/sequential steroid monotherapy	Dialysis dependence
4^[12]^	50/F	History of recurrent tonsillitis	232.0	15/HPF	0.41 g/24 h	Normal	Normal sized kidneys	89%	Linear capillary loop staining for IgG (3+) together with mesangial staining for IgA (4+), IgM (1+), and C3 (2+)	258.3 U/mL	Pulse dose of intravenous methylprednisolone/sequential steroid combined with MMF	Creatinine rechecked was 74 mmol/L after 20 mo
5^[13]^	66/F	IgA nephropathy	400.4	100/HPF	NR	NR	NR	72%	Linear capillary loop staining for IgG and C3 together with mesangial staining for IgA and C3	116 U/mL	Pulse dose of intravenous methylprednisolone/plasma exchange/sequential steroid monotherapy	Dialysis dependence
6^[14]^	22/M	No	77.0	250/HPF	0.5 g/24 h	Bilateral lower lobe patchy heterogeneous parenchymal opacities	NR	18%	Linear capillary loop staining for IgG and C3 together with mesangial staining for IgA	Positive	Plasma exchange/sequential steroid combined with cyclophosphamide/sequential steroid combined with azathioprine 2 mo later	Serum creatinine remained within the normal range after 4 mo
7^[15]^	38/F	No	481.8	Gross hematuria	3.5 g/24 h	Normal	NR	69%	Linear capillary loop staining for IgG and granular deposition of IgA in mesangial spaces	187.2 U/mL	Pulse dose of intravenous methylprednisolone and cyclophosphamide/sequential steroid monotherapy	Serum creatinine decreased to 183.9 μmol/L after 3 mo
8^[16]^	66/F	Left partial nephrectomy	320.0	3+	0.77 g/24 h	Normal	NR	55%	Linear capillary loop staining for IgG (3+) together with mesangial staining for IgA (2-3+)	79 U/mL	Pulse dose of intravenous methylprednisolone/plasma exchange/sequential steroid combined with cyclophosphamide	Serum creatinine decreased to 180 μmol/L after 12 mo
9^[17]^	Old/F	After SARS-CoV-2 mRNA vaccination	689.5	Gross hematuria	NR	Normal	NR	100%	Linear capillary loop staining for IgG (3+) together with mesangial staining for IgA (2-3+)	Positive	Treated with methylprednisolone, cyclophosphamide, plasmapheresis and hemodialysis	Dialysis dependence
10^[18]^	22/F	No	282.9	3+	3+	Normal	Normal sized kidneys	70%	Linear capillary loop staining for IgG (2+) together with mesangial staining for IgA (3+) and C3 (1+)	96 U/mL	Pulse dose of intravenous methylprednisolone/sequential steroid monotherapy	Non dialysis dependent chronic renal failure
11^[19]^	54/M	Renal transplant	720.0	4+	2+	NR	NR	NR	Linear capillary loop staining for IgG, Kappa and Lambda together with mesangial staining for IgA and C3	Positive	Pulse dose of intravenous methylprednisolone and cyclophosphamide/plasma exchange	Died from sepsis
12^[20]^	38/F	History of upper respiratory tract infection before the disease	503.9	10/HPF	2.2 g/24 h	Normal	Normal sized kidneys	28%	Linear capillary loop staining for IgG, finely granular capillary loop staining for IgA together with mesangial staining for IgA and C3	237.6 U/mL	Pulse dose of intravenous methylprednisolone and cyclophosphamide/sequential steroid monotherapy	Serum creatinine decreased to 194.5 μmol/L after 4 mo
13^[21]^	41/F	History of upper respiratory tract infection before the disease	278.3	476.69/HPF	2.1 g/24 h	Bilateral pleural effusion, local atelectasis, and chronic inflammation	NR	NR	Linear capillary loop staining for IgG together with mesangial staining for IgA	47.2 U/mL	Pulse dose of intravenous methylprednisolone/rituximab/plasma exchange/intravenous immunoglobulin/sequential steroid combined with tacrolimus	Serum creatinine decreased to 151.7 μmol/L after 28 wks with hematuria and proteinuria improved significantly
14^[22]^	27/M	History of upper respiratory tract infection before the disease	1347.0	Gross hematuria	NR	NR	NR	100%	Granular deposits of IgA +++ and IgG + along glomerular capillary walls and in mesangium	Positive	NR	Dialysis dependence
15^[23]^	55/M	Type 1 diabetes mellitus/HIV infection/resistant MRSA septic	309.4	772/HPF	2+	Normal	Mildly enlarged kidneys	10%	Linear capillary loop staining for IgG together with mesangial weak granular staining for IgA	8.6 U/mL	Rituximab/intravenous immunoglobulin/sequential steroid combined with MMF	Serum creatinine decreased to 106.1 μmol/L after 16 mo
16(this case)	26/F	History of upper respiratory tract infection before the disease	174.1	1032.4/HPF	10.2 g/24 h	Normal	Normal sized kidneys	48%	Linear capillary loop staining for IgG (3+) and C3 (2+) together with mesangial granular staining for IgA (3+)	142 U/mL	Pulse dose of intravenous methylprednisolone and cyclophosphamide e/plasma exchange/sequential steroid monotherapy	Serum creatinine decreased to 71 μmol/L after 1 yr

CT = computed tomography, F = female, M = male, MRSA = methicillin-resistant staphylococcus aureus, NR = not reported, RBC = red blood cell, GBM = glomerular basement membrane, HBV = hepatitis B virus, HIV = human immunodeficiency virus, HPF = high-power field, IgA = immunoglobulin A, SARS-CoV = severe acute respiratory syndrome coronavirus.

In these concurrent anti-GBM disease and IgA nephropathy cases, the average age is 40 years, which seems to be consistent with the classic bimodal distribution of anti-GBM disease.^[24]^ Interestingly, the male to female ratio is 1:2, which is inconsistent with the current finding that both anti-GBM disease and IgA nephropathy are highly prevalent in men.^[25,26]^ Estrogen regulates a variety of cytokines and influences the development and function of B and T cells, resulting in the regulation of inflammatory responses.^[27]^ However, the relationship between estrogen and anti-GBM has rarely been reported. Despite the heavier clinicopathological manifestations in male IgA nephropathy patients,^[28]^ 1 study found that castration of mice increased the severity of VT-induced IgA nephropathy, but supplementation with estrogen did not diminish this effect but increased the severity of the disease.^[29]^ In addition, a bioinformatic study based on the Gene Expression Omnibus database found that many key genes upregulated in IgA nephropathy are associated with the estrogen signaling pathway.^[30]^ Therefore, it is speculated that estrogen may play an important role in the development of concurrent anti-GBM disease and IgA nephropathy; however, further studies are required.

In terms of population distribution, these cases were reported in China (n = 7), Japan (n = 2), the United States (n = 2), Australia (n = 2, 1 Asian, 1 Caucasian), Korea (n = 1), Canada (n = 1), and India (n = 1). 75% of them were from Asia, and mainly from East Asia. Taken together, this may reflect the large regional and ethnic variation in the prevalence of IgA nephropathy.^[31]^ It has been reported that IgA nephropathy accounts for 30% to 50% of kidney biopsy patients in Asia and the Pacific, about 20% in Europe, and only about 10% in North America.^[32]^ In North American, there is also a significant difference in the prevalence of IgA nephropathy between native North Americans (38%) and African Americans (2%), supporting the ethnic variation.^[33,34]^ However, the causal relationship between anti-GBM disease and IgA nephropathy is still unclear. It is well known that IgA nephropathy has various clinical symptoms, ranging from asymptomatic microscopic hematuria to nephrotic syndrome and even RPGN.^[35]^ All these patients presented with hematuria and 75% of them presented with gross hematuria. Including the case we reported, there were 4 patients had a history of upper respiratory tract infection before the onset of the disease. In addition, Tadasu et al reported a case of anti-GBM disease that occurred during the course of IgA nephropathy.^[13]^ These suggested a possibility that anti-GBM disease was superimposed on IgA nephropathy and that IgA nephropathy may be underlying. One hypothesis is that IgA-associated immune complexes may promote immunological and inflammatory events that lead to conformational changes in GBM and antigens exposure, resulting in the production of anti-GBM antibodies.^[19]^ It has also been hypothesized that deposition of aberrant IgA along the GBM in IgA nephropathy may induce the formation of novel antigens, leading to the production of anti-GBM antibodies.^[22]^ However, since no biomarkers have been identified to distinguish primary from secondary anti-GBM disease, it is difficult to prove whether anti-GBM disease in these patients is an incidental complication or secondary to IgA nephropathy. Another hypothesis proposes that anti-GBM antibodies may alter the permeability of GBM, allowing the deposition of circulating immune complexes in the mesangium.^[22]^ The number of relevant cases was too small, making these hypotheses difficult to prove. Furthermore, owing to the severity and rapidity of anti-GBM disease, the number of undamaged glomeruli is very limited. Therefore, the pathological features of IgA nephropathy may not be observed, possibly resulting in some patients having anti-GBM disease accompanied by IgA nephropathy not being reported.

Since anti-GBM disease progresses rapidly and its prognosis is closely related to the severity of the disease at the start of treatment, a combination of plasmapheresis and immunosuppression should be initiated without delay to render the anti-GBM antibody titer to negative as soon as possible.^[36]^ Plasma exchange is recommended for all patients with anti-GBM disease, except those with 100% glomerular crescents and no pulmonary hemorrhage.^[37]^ In addition, plasma exchange should be continued until anti-GBM titers are undetectable.^[38]^ Glucocorticoids combined with cyclophosphamide is the preferred standard treatment. Rituximab has shown promising effects in refractory anti-GBM disease^[39]^; however, a retrospective study with a small sample size found no effect on renal remission in anti-GBM disease patients when rituximab was substituted for cyclophosphamide as a first-line agent.^[40]^ Successful use of mycophenolate or mycophenolic acid instead of cyclophosphamide in the treatment of anti-GBM disease has also been reported.^[41,42]^ Experience in the treatment of concurrent anti-GBM disease and IgA nephropathy is obviously even less. In the cases we reviewed, except for 1 lacking treatment information, corticosteroids were used in all cases, in 60% of cases plasmapheresis was applied, in 60% of cases cyclophosphamide was used, only 2 cases applied mycophenolate mofetil and 2 cases applied rituximab. The main reason why patients did not undergo a standard treatment strategy was the concern about the side effects of cyclophosphamide and plasmapheresis. In terms of prognosis, 56.3% of cases showed improved renal function and 31.3% showed dialysis dependence. Interestingly, none of the patients treated with mycophenolate mofetil or rituximab developed dialysis dependence, suggesting that they may have potential in the treatment of concurrent anti-GBM disease and IgA nephropathy.^[12,21,23]^ Notably, all patients who eventually depended on dialysis had higher creatinine levels when they started immunosuppressive therapy, the lowest of which was 400.4 μmmol/L. In contrast, all patients with a good prognosis had low serum creatinine levels, below 320 μmmol/L except for 1 case of 481.8 μmmol/L. This strongly suggests that the timely administration of immunosuppressive therapy has a significant impact on the prognosis of concurrent anti-GBM disease and IgA nephropathy. The largest histopathological study of anti-GBM disease with a median follow-up of 3.9 years in 123 patients also found that patients with creatinine levels below 500 mmol had a better prognosis, and they reported an overall dialysis-dependent rate of 69% for anti-GBM disease, much greater than the 31.3% for concurrent anti-GBM disease and IgA nephropathy reported in our review,^[26]^ indicating that concurrent anti-GBM disease and IgA nephropathy may have a tendency for better prognosis.

Interestingly, the patient we reported had a 24-hour urinary protein quantification of 10.2 g. Such high levels of proteinuria are uncommon in both IgA nephropathy and anti-GBM diseases. We speculate that this may be related to the presence of extensive podocyte effacement. Podocyte effacement can be observed in some IgA nephropathy cases, and its severity is positively correlated with the patient’s proteinuria levels.^[43,44]^ Podocyte effacement has rarely been reported in classic anti-GBM diseases. However, Liang et al reported that many patients with antibody-negative anti-GBM disease had global podocyte effacement and often accompanied with nephrotic-range proteinuria.^[45]^ In the cases we reviewed, only 1 patient was reported to have extensive podocyte effacement and this patient also had a high urinary protein of 3.76 g/24 h.^[9]^ The mechanism underlying extensive podocyte effacement in IgA nephropathy and anti-GBM disease requires investigation.

The patient we report had not only a detailed history of treatment, but also complete follow-up information. In the early stage of the disease, the patient refused immunosuppressive therapy owing to the normal creatinine level and personal considerations of the side effects of immunosuppression. However, as the condition worsened, corticosteroids and cyclophosphamide in combination with plasmapheresis were administered with the patient’s consent, and the patient achieved a very good prognosis with complete normalization of renal function and complete disappearance of hematuria and proteinuria at subsequent follow-up. By reviewing all concurrent anti-GBM disease and IgA nephropathy cases, the good prognosis of this case may be mainly related to the relatively timely treatment and the potential tendency for better prognosis of the disease itself. Because of the limited number of reported cases and variable follow-up time, more cases need to be collected to better understand the clinical, pathological, and prognostic information of concurrent anti-GBM disease and IgA nephropathy.

## 4. Conclusion

Concurrent anti-GBM disease and IgA nephropathy remains a very aggressive and rapidly progressive disease. Early pathologic diagnosis and timely immunosuppressive therapy are the key to a good prognosis. In addition, concurrent anti-GBM disease and IgA nephropathy may have a tendency for better prognosis than classic anti-GBM disease.

## Author contributions

FS, SS, and HJ prepared the manuscript’s first draft. WL, YJ and ZF retrieved and corroborated the data. XZ prepared the figures and edited the manuscript. WH contributed to manuscript revision, read, and approved the submitted version.

Conceptualization: Su Sensen.

Data curation: Wang Luyu, Zhang Fei, Yu Jinyu.

Funding acquisition: Wu Hao.

Writing – original draft: Fu Shaojie, Huang Jingda.

Writing – review & editing: Xu Zhonggao, Wu Hao.
